# Mycotoxin Interactions along the Gastrointestinal Tract: In Vitro Semi-Dynamic Digestion and Static Colonic Fermentation of a Contaminated Meal

**DOI:** 10.3390/toxins14010028

**Published:** 2022-01-01

**Authors:** Maria Madalena Costa Sobral, Tiago Gonçalves, Zita E. Martins, Christine Bäuerl, Erika Cortés-Macías, Maria Carmen Collado, Isabel M. P. L. V. O. Ferreira

**Affiliations:** 1 LAQV/REQUIMTE—Laboratório de Bromatologia e Hidrologia, Faculdade de Farmácia da Universidade do Porto, 4050-313 Porto, Portugal; dtiago.tg@gmail.com (T.G.); zmartins@ff.up.pt (Z.E.M.); 2 IATA-CSIC—Institute of Agrochemistry and Food Science-National Research Council, Agustín Escardino 7, 46980 Valencia, Spain; christine.bauerl@iata.csic.es (C.B.); ercorma@iata.csic.es (E.C.-M.); mcolam@iata.csic.es (M.C.C.)

**Keywords:** aflatoxin B1, ochratoxin A, gut microbiota, inflammation, in vitro models

## Abstract

Aflatoxin B1 (AFB1) and ochratoxin A (OTA) naturally co-occur in several foods, but no studies have followed the fate of mycotoxins’ interactions along the gastrointestinal tract using in vitro digestion models. This study used a novel semi-dynamic model that mimics gradual acidification and gastric emptying, coupled with a static colonic fermentation phase, in order to monitor mycotoxins’ bioaccessibility by the oral route. AFB1 and OTA bioaccessibility patterns differed in single or co-exposed scenarios. When co-exposed (MIX meal), AFB1 bioaccessibility at the intestinal level increased by ~16%, while OTA bioaccessibility decreased by ~20%. Additionally, a significant increase was observed in both intestinal cell viability and NO production. With regard to mycotoxin–probiotic interactions, the MIX meal showed a null effect on *Lactobacillus* and *Bifidobacterium* strain growth, while isolated AFB1 reduced bacterial growth parameters. These results were confirmed at phylum and family levels using a gut microbiota approach. After colonic fermentation, the fecal supernatant did not trigger the NF-kB activation pathway, indicating reduced toxicity of mycotoxins. In conclusion, if single exposed, AFB1 will have a significant impact on intestinal viability and probiotic growth, while OTA will mostly trigger NO production; in a co-exposure situation, both intestinal viability and inflammation will be affected, but the impact on probiotic growth will be neglected.

## 1. Introduction

Mycotoxins are toxic metabolites usually found in several food products as a result of fungi spoilage. Aflatoxin B1 (AFB1) and ochratoxin A (OTA) are mycotoxins with recognized human toxicity, being classified as carcinogenic (group 1) and possibly carcinogenic (group 2B) to humans, respectively, by the International Agency for Research on Cancer (IARC) [1]. AFB1 and OTA are prevalent mycotoxins found in a wide range of food products, including cereals and dairy products [2,3,4]. In addition, as these two mycotoxins co-occur naturally in several foods [5,6,7] and as meals combine food products with distinct origins, multiple-mycotoxin contamination is a real situation. Indeed, the European Food Consumption Validation (EFCOVAL) project recently revealed that 600 individuals from five European countries were exposed to mixtures of mycotoxins or their metabolized forms by assessing their content in urine and serum [8]. AFB1 and OTA were among the detected mycotoxins at concentrations ranging from 0.001 to 10 ng/L and 0.1 to 100 ng/L, respectively, in urine and serum samples.

Understanding the fate of contaminants along the gastrointestinal tract (GIT) and interactions with nutrients is a challenge. During the past decade, in vitro digestion methodologies have facilitated the simulation of in vivo conditions. The international INFOGEST network published two distinct in vitro methodologies. The first, in 2014, is a static approach that mimics the physiological conditions of human digestion. This recommends the use of a constant ratio between food and enzyme/electrolytes and a constant pH, time, and temperature [9]. Although useful, it lacks other relevant physiological conditions, such as the gradual acidification of the stomach and gastric emptying [10]. Thus, in 2020, a standardized semi-dynamic digestion model was published [10], considering also the energy value of food/meal for the simulation of the gastric phase, since it impacts the time of residence in the stomach.

No studies have been found concerning the release of mycotoxins from the stomach to the duodenum nor the impact of the gradual acidification on their matrix release. Thus, this study introduces the first approach to using a semi-dynamic model to evaluate AFB1 and OTA release during GI digestion, as well as mycotoxin–food interactions. Moreover, AFB1 and OTA bioaccessibility is also influenced by their water solubility. AFB1 and OTA have a lipophilic character with a coefficient of partitioning of 1.23 and 4.74, respectively, which suggests that part of these mycotoxins will not become bioaccessible during GI digestion, reaching the colon. In addition, some food constituents (i.e., fibers, proteins) have shown to impact mycotoxins’ bioaccessibility [11]. Concerning the bioaccessibility of mycotoxins along the GIT, the few studies published over the past years focus on a specific food matrix (e.g., meat, cereals, fish) [5,12,13], with only two studies evaluating mycotoxins’ bioaccessibility using a meal approach [14,15]. However, none of these reports explores the interactions of mycotoxins after ingestion, and no information has been found regarding AFB1 and OTA interactions during the digestion process of a snack meal.

Beyond digesting foods and allowing nutrient uptake, the GIT also plays an important role in the immune system, which can be compromised by mycotoxins’ presence, as these have shown to damage the intestinal epithelium and contribute to oxidative stress [16]. AFB1 and OTA are known to increase the permeability of the epithelial barrier and decrease epithelial cells’ viability [16]; these mycotoxins have also been shown to modulate the host’s immune system by suppressing immune functions: (i) AFB1 alone or together with other AFs has been shown to reduce reactive oxygen species (ROS) and nitric oxide (NO) production; (ii) in the same way, OTA reduced the gene expression of nuclear factor kB (NF-kB) and several cytokines in the duodenum after piglets’ 30-day exposure to sub-chronic OTA levels (0.05 mg/kg feed) [17].

The gut microbiota plays a crucial role in human health by maintaining immune and metabolic homeostasis; however, some external factors, including exposure to xenobiotics, may imbalance the gut bacteria composition, leading to unhealthy outcomes [18,19]. In fact, it is believed that continuous exposure to toxins benefits the predominance of harmful bacteria over beneficial ones, which may contribute to the development of several disorders, such as obesity, autoimmune diseases, and infections [20]. Exposure of the gut microbiota to mycotoxins may trigger two different scenarios: (i) modulation of the gut microbiota profile due to mycotoxins’ antimicrobial properties or toxic effects toward bacteria or (ii) changing of mycotoxins’ toxicity (increasing or decreasing) as result of degradation/metabolization by some fecal bacteria [21]. Several animal studies have shown that AFB1 and OTA are able to change the microbiota profile, having a significant impact on taxonomic abundance at family and genus levels, as reviewed by Guerre [21]. Specifically, exposure of mice to OTA caused marked changes in gut microbial diversity, decreasing the relative abundance of Firmicutes at the phylum level [22]. In contrast, *Lactobacillus casei* Shirota was suggested as a detoxifying player when treating rats [23], given their ability to reduce AFB1 toxicity. In contrast, Wang et al. [24] suggested that AFB1 could alter the gut microbiota in a dose-dependent manner, decreasing phylogenic diversity.

The literature shows studies focused on gastric and duodenal bioaccessibility of mycotoxins using in vitro static methods and on numerous food matrices [5,12,15,25,26,27], but the fate of mycotoxins by simulating GI digestion of a contaminated snack meal has not been assessed. Moreover, mycotoxin–microbiota interaction studies found in the literature are animal-based studies and do not relate to the human gut microbiota [21]. An in vitro semi-dynamic digestion methodology followed by static in vitro colonic fermentation was used to assess (i) the bioaccessibility of isolated AFB1 and OTA or as a mixture during GI digestion of a meal, monitoring their release from the stomach to the duodenum through gastric emptying simulations; (ii) the impact of non-bioaccessible mycotoxins on important probiotics; and (iii) the impact of both bioaccessible and non-bioaccessible mycotoxins on intestinal epithelium viability and inflammation.

## 2. Results

### 2.1. Mycotoxins’ Analytical Method Performance

The matrix had a significant effect (>20% or <−20%) on the analytes’ quantification, either enhancing or decreasing their analytical response. The percentage of matrix effect measured according to SANTE guidelines [28] (see Section 4.3) was 71% for AFB1 and 56% for OTA. Therefore, the linearity of the chromatographic method was determined by preparing matrix-matched calibration curves using five points at amounts ranging from 2 to 40 ng in the meal and 1 to 20 ng in digested matrices (Table 1). A good linear response was obtained in all matrices (R^2^ > 0.993).

The recoveries of mycotoxins were within recommended values (80–120%) at all tested concentrations, with relative standard deviations (% RSD) lower than 25% (Table 2), except for OTA at the highest spiked level (55 ng/mL).

The validated method was then applied to confirm the absence of mycotoxins in the meal, before artificial contamination, and to follow the mycotoxins’ fate and interactions along the gastrointestinal tract.

### 2.2. Mycotoxins Bioaccessibility during Gastrointestinal Digestion

**Figure 1:**
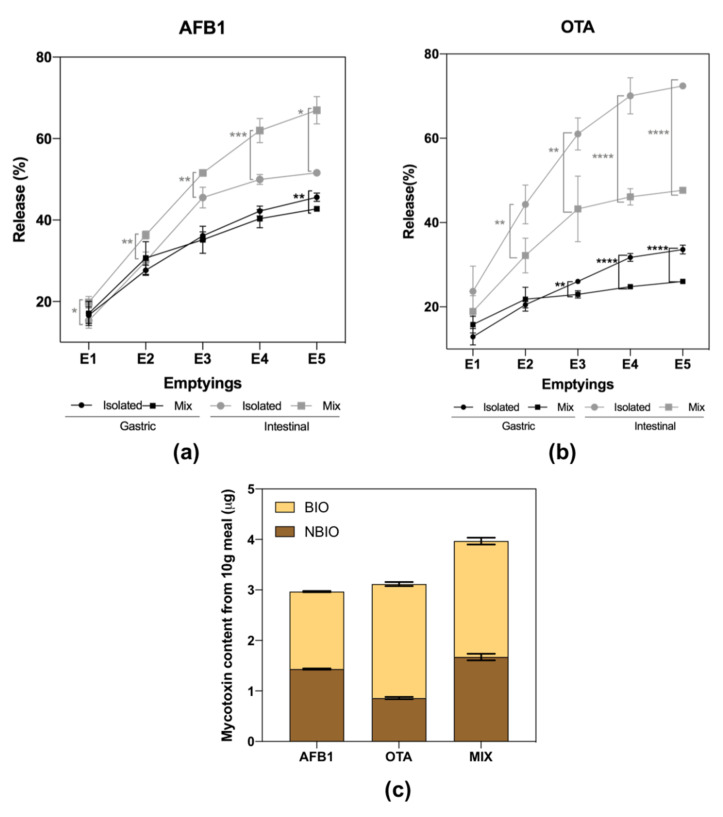
Bioaccessibility of AFB1 (**a**) and OTA (**b**) during semi-dynamic gastro-duodenal digestion. The graphs represent the cumulative release of AFB1 and OTA in each gastric emptying and respective duodenal digestion when digested isolated or as a mixture. (**c**) Mycotoxins’ distribution along intestinal emptying (IE1 to IE5) and the content that remained in the non-bioaccessible fraction (NBIO). Data are presented as the mean ± SD of four replicates (*n* = 4). Parametric (*t*-test) or non-parametric (Mann–Whitney) statistical tests were applied to compare AFB1 or OTA release, isolated or in combination, and statistical differences are indicated as * *p* < 0.05, ** *p* < 0.01, *** *p* < 0.001, and **** *p* < 0.0001. E1 to E5 mean emptying 1 to emptying 5.

Isolated AFB1 and OTA were released along GI digestion, exhibiting a maximum cumulative isolated bioaccessibility of 51.6 and 72.4%, respectively, after intestinal digestion (Figure 1a,b). Moreover, the earlier gastric emptyings (GEs; E1 to E3) had the greatest contribution to AFB1 and OTA release into the duodenum, meaning that the time of residence of these mycotoxins in the stomach is short (~30 min), becoming quickly available for absorption or for exerting their toxic effects in the intestine. Figure 1c shows the mycotoxins’ distribution concerning the final content that became bioaccessible in the intestinal phase (BIO fraction) and the content that remained non-bioaccessible (NBIO fraction) in relation to its initial content in 10 g of meal.

Regarding interactions, the release of AFB1 or OTA was significantly affected by the presence of the other mycotoxin. The digestion of a meal contaminated with both mycotoxins promoted a release of AFB1 over the gastric phase significantly (*p* < 0.01) lower (41.5%) than its isolated bioaccessibility (45.5%). However, in the intestinal phase, AFB1 exhibited significantly (*p* < 0.01) higher final intestinal bioaccessibility in the meal contaminated with both mycotoxins (67.3%) when compared to its isolated bioaccessibility (51.6%). In contrast, OTA’s bioaccessibility was lower in the meal contaminated with both mycotoxins: in the gastric phase, the bioaccessibility of isolated OTA was 33.6% vs. 27.2% in the meal that contained both mycotoxins. The intestinal bioaccessibility of OTA in all gastric emptyings was significantly lower in the meal contaminated with both mycotoxins, exhibiting a maximum bioaccessibility of 52.8% when its isolated bioaccessibility was 72.4%. Thus, meals co-contaminated with AFB1 and OTA will likely increase AFB1 bioaccessibility by ~16% and decrease OTA bioaccessibility by ~20% at the intestinal level.

### 2.3. Impact of Bioaccessible Mycotoxins on Intestinal Cells’ Viability and Inflammation

**Figure 2:**
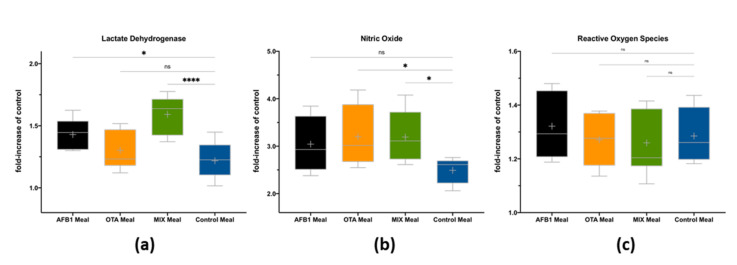
Impact of bioaccessible mycotoxins on lactate dehydrogenase release (**a**), nitric oxide formation (**b**), and reactive oxygen species formation (**c**) after 3 h exposure of 7-day-differentiated Caco-2 cells. Digests were 12× diluted prior to exposure. Data represent the fold-increase related to cells’ control and are presented as the mean ± SD of two independent experiments. Parametric (Dunnett’s post hoc test) or non-parametric (Kruskal–Wallis and Dunn’s post hoc) tests were applied to compare the effects of AFB1, OTA, or MIX meals with those of the control meal (without mycotoxins). Statistical differences are indicated as * *p* < 0.05 and **** *p* < 0.0001.

There is an impact of BIO fractions after 3 h exposure of 7-day-differentiated Caco-2 monolayers on cell viability and the formation of nitric oxide and reactive oxygen species (Figure 2).

When compared to the control meal, the digested samples contaminated with AFB1 or both mycotoxins (MIX) significantly increased cell death (Figure 2a) by releasing lactate dehydrogenase from the cytosol into the culture medium, while at the studied doses, bioaccessible OTA did not present a significant impact on cells’ viability. These results were corroborated by strong Pearson correlations (r > 0.6) between LDH measurements in Caco-2 cells and AFB1 contents in the meal before digestion and in the bioaccessible fraction (Table 3).

Nitric oxide formation only differed from the control meal (*p* < 0.05) in digested meals with OTA or MIX (Figure 2b). These results could be supported by a moderate Pearson correlation (r = 0.535) between NO and OTA content in the meal; however, this correlation was not observed between NO and bioaccessible OTA. When summing up AFB1 and OTA bioaccessible contents, moderate correlations (r > 0.525 and r > 0.446) were observed between AFB1 + OTA bioaccessible content and NO and LDH, respectively (Table 3). The bioaccessible mycotoxins did not impact reactive oxygen species formation (Figure 2c).

### 2.4. Impact of Non-Bioaccessible Mycotoxins on Probiotic Lactobacillus casei and Bifidobacterium lactis

**Figure 3:**
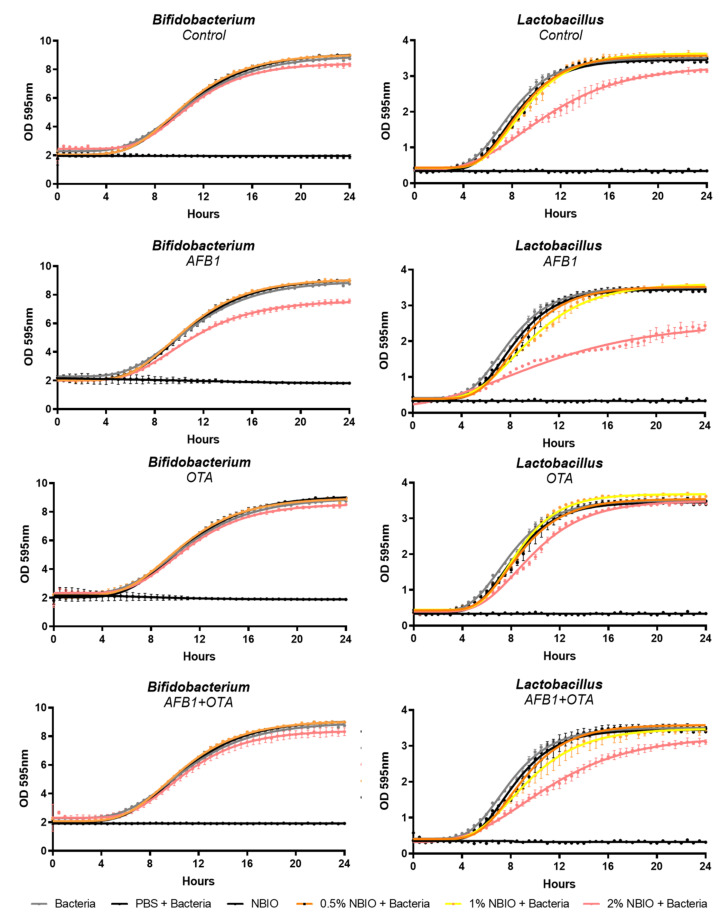
Impact of non-bioaccessible mycotoxins on Bifidobacterium lactis and Lactobacillus casei growth over 24 h incubation at anaerobic conditions. Data are presented as the mean ± SEM of four replicates (*n* = 4). PBS—phosphate buffer solution; NBIO—non-bioaccessible fraction.

The impact of different amounts of non-bioaccessible (NBIO) fraction (0.5%, 1%, and 2%) on two important probiotic strains (*Lactobacillus casei* BL23 and *Bifidobacterium lactis* NCC 2818) was evaluated to understand how non-bioaccessible mycotoxins affect bacterial growth and to select the best NBIO percentage for colonic fermentation assays. Data are presented in Figure 3 and Table 4.

The NBIO fraction with AFB1, OTA, or MIX at 0.5% did not exert any significant changes in relation to the control, except for the maximal optical density (MOD) of the *Bifidobacterium* strain in the NBIO fraction containing AFB1 or OTA. The increase of NBIO fractions to 1% or 2% significantly impacted bacterial growth parameters: at 1%, both bacterial growth and lag time of the *Lactobacillus* strain significantly reduced in the presence of AFB1 and MIX, while OTA only affected (increased) the growth rate; with regard to the *Bifidobacterium* strain, only the growth rate and MOD were affected by AFB1 and OTA, while MIX did not differ from the control.

The exposure of both bacterial strains to 2% NBIO containing AFB1 or OTA significantly impacted all growth parameters in relation to the control, except for OTA in the case of the growth rate of *Bifidobacterium*. The mycotoxin type differently impacted bacterial growth: (i) AFB1 significantly reduced the measured parameters, (ii) OTA significantly increased them, while (iii) MIX had a null effect on bacterial growth.

Pearson correlations between the mycotoxin content in NBIO fractions and bacterial growth parameters showed the same opposite behavior between mycotoxins (Table 5):

(i) Strong negative Pearson correlations between the AFB1 content in the meal and all growth parameters of both *L. casei* and *B. lactis*, suggesting a negative impact on bacterial growth (i.e., reduction in the MOD, growth rate, and lag time). Even stronger negative correlations were observed between the non-bioaccessible AFB1 content and probiotic growth parameters, indicating an inhibition of growth in both strains by AFB1.
(ii) Strong positive correlations between the OTA content in the meal and *L. casei* growth parameters and the *B. lactis* MOD. Moderate correlations were observed between the non-bioacessible OTA content and *L. casei* growth, confirming an enhancement of the growth rate.
(iii) No correlation was found between bacterial growth and mycotoxin content when summing up their contents (AFB1 + OTA), suggesting a null effect in the co-exposure situation.

Given that the NBIO fraction at 2% showed the greatest impact on bacterial growth, this percentage was selected for colonic fermentation assays.

### 2.5. Impact of Non-Bioaccessible Mycotoxins on the Gut Microbiota, Intestinal Cells’ Viability, and Inflammation

**Figure 4:**
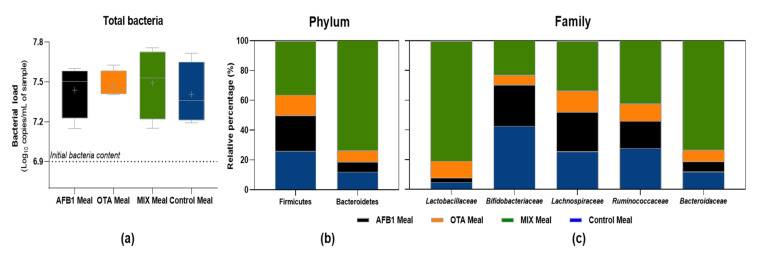
Impact of non-bioaccessible mycotoxins on gut microbiota growth over 24 h incubation under anaerobic conditions: (**a**) total bacteria, (**b**) at the phylum level (Firmicutes and Bacteriodetes), and (**c**) at the family level (*Lactobacillaceae, Bifidobacteriaceae, Lachnospiraceae, Ruminococcaceae*, and *Bacteroidaceae*). Data for total bacteria are presented as the mean ± SD of four replicates (*n* = 4).

After colonic fermentation, an increase in the total bacteria content was observed (Figure 4a), but the presence of AFB1, OTA, or MIX did not influence the total bacteria content. However, mycotoxins were able to change the gut microbiota composition at the phylum and family levels. At the phylum level (Figure 4b), MIX meals followed by control meals were the samples that had the highest percentage of Firmicutes and Bacteroidetes, while AFB1 and OTA meals revealed lower abundances. At the family level (Figure 4c), the MIX meal had the highest percentage of *Bacteroidaceae*, *Ruminococcaceae*, and *Lachnospiraceae*, followed by control, AFB1, and OTA meals, respectively. Differently, *Lactobacillaceae* exhibited higher percentages in the MIX meals, followed by OTA meals, with AFB1 and control meals having a lesser contribution. With regard to *Bifidobacteriaceae*, the control meal had the highest contribution, followed by AFB1 and MIX meals, with OTA meals having the lower abundances.

**Figure 5:**
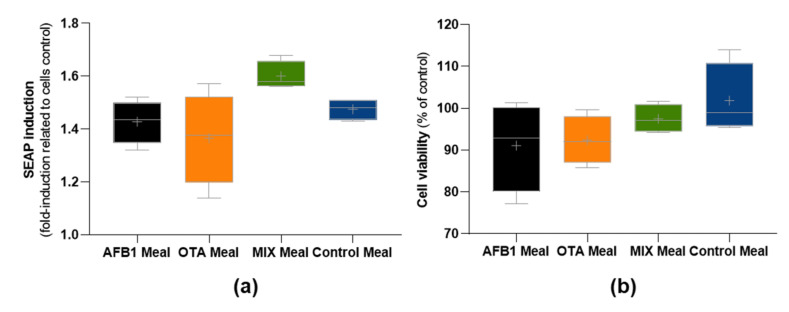
Impact of non-bioaccessible mycotoxins on secreted embryonic alkaline phosphatase (SEAP) activity (**a**) and cell viability (**b**) after 24 h exposure of colonic HT29 reporter cells monitoring NF-kB activity. Digests were 10× diluted prior to exposure. Data represent the fold-increase related to the control (only cells) and are presented as the mean ± SD of two independent experiments.

Regarding the impact of NBIO fractions on intestinal cells’ viability and inflammation, the NBIO samples before colonic fermentation and the fecal supernatant after colonic fermentation did not significantly trigger an NF-kB inflammatory response on human colon HT29 reporter cells (Figure 5a,b).

## 3. Discussion

Mycotoxin bioaccessibility values represent the maximum amount that can be absorbed, which can be interpreted as the worst-case scenario of humans’ exposure. These are relevant data regarding mycotoxins’ risk assessment determination; thus, reliable models of the digestive process are used to obtain more accurate and precise information. Herein, a novel semi-dynamic digestion model, followed by static colonic fermentation, was used to understand the mycotoxins’ behavior along the GIT [10]. To the best of our knowledge, this is the first approach combining supervision of mycotoxin release from the stomach to the duodenum, together with the evaluation of the impact of non-bioaccessible mycotoxins on important gut probiotics. Moreover, this study also aimed at assessing the mycotoxins’ fate alone and in a co-exposure situation along the GIT and gain more insights into mycotoxins’ impact on intestinal epithelium viability and inflammation in intestinal and colonic phases.

The final intestinal bioaccessibility of AFB1 or OTA was affected by the presence of the other mycotoxin, increasing AFB1 release and reducing OTA’s. These interactions have already been reported between OTA and patulin during digestion, increasing their bioaccessibility compared to their single-exposure situation [5]. The promotion of AFB1 bioaccessibility represents a risk, since AFB1 belongs to the most dangerous mycotoxins in terms of carcinogenic effects [1]. In addition, the short time of residence in the stomach (~30 min) observed for both mycotoxins indicates fast availability in the duodenum to exert their toxic effects on the intestinal epithelium or on other target organs after absorption (e.g., liver and kidney) [1]. The co-ocurrence of AFB1 and OTA is a real situation with regard to several foods products, including dairy products and cereals [2,7], but limited data are found on their combined bioaccessibility: (i) Kabak et al. [27] reported 90% and 30% bioaccessibility for AFB1 and OTA, respectively, in several food matrices spiked or naturally contaminated, and (ii) Versantvoort et al. [15] reported bioaccessibility above 100% for both mycotoxins after digesting peanut slurry. These results differ from those obtained in this study, probably due to the food matrix itself that has a relevant influence on compounds’ release during digestion and the use of a different in vitro model, as Kabak et al. and Versantvoort et al. used a non-standardized in vitro static digestion model [15,27]. The use of standardized methodologies is of paramount importance to allow comparison of experiments among different research groups, as several parameters may affect the results. For example, the gradual acidification of the stomach affects protein structures and interactions with matrix constituents and/or other compounds [10], which may influence mycotoxin bioaccessibility. Indeed, AFB1 and OTA have a high capacity to establish covalent bonds with proteins [29]. The gradual acidification observed in this study agrees with previous results reported during digestion of semi-solid dairy products [10].

The intestinal epithelium represents the first barrier met by xenobiotics, such as dietary mycotoxins, known to cause several GI disorders [30,31]. Herein, AFB1 and OTA had different effects on intestinal viability and inflammation when compared to the control meal: (i) the digested meal containing AFB1 significantly reduced intestinal cells’ viability, but no effect was observed on inflammation; (ii) the digested meal with OTA did not impact cells’ viability but had a pro-inflammatory effect, increasing NO production; (iii) the meal containing both mycotoxins (MIX) significantly impacted both intestinal cells’ viability and NO production; and (iv) none of the meal samples containing mycotoxins triggered ROS formation. These data are related with each mycotoxin content in the meal or in the bioaccessible fraction, as confirmed by the strong or moderate Pearson correlations obtained for AFB1, OTA, and AFB1 + OTA contents. Thus, the increased LDH values observed in digested AFB1 and MIX meals are explained by the AFB1 content and its recognized intestinal toxicity [32,33]. Therefore, the higher LDH formation in the digested MIX meal is explained by the higher AFB1 content as a result of increased bioaccessibility in the MIX meal. The disruption of the intestinal barrier permeability of Caco-2 cells (reduced cell viability and tight-junction protein expression) was observed after exposure to increasing concentrations of AFB1 (3 to 100 µM) and OTA (1 to 100 µM) [32]. In the case of NO production, bioaccessible OTA and MIX triggered similar pro-inflammatory responses, which could suggest an interaction between AFB1 and OTA, since OTA had lower intestinal bioaccessibility (52.8%) in MIX when compared to the isolated bioaccessibility (72.4%).

Altogether, in a meal with single-mycotoxin contamination (AFB1 or OTA), only 50% of AFB1 will be bioaccessible and the remaining content will follow colonic fermentation, whereas for OTA, more than 70% will become bioaccessible. The type of mycotoxins present in the meal will likely cause different intestinal health impacts. However, in a co-exposure situation, there will be a promotion of AFB1 bioaccessibility up to 70% and OTA reduction of about 50%, tending to cause both loss of intestinal viability and increase in NO production. This means that the amount of AFB1 or OTA that will undergo colonic fermentation is dependent on the presence of other mycotoxins.

Herein, a considerable amount (32.7 to 48.4% of AFB1 and 27.6 to 47.2% of OTA) remained in the non-bioaccessible fraction, representing a different threat to the gut microbiota. AFB1 had a greater impact than OTA on both *Lactobacillus casei* and *Bifidobacterium lactis* growth curve parameters, while MIX exhibited a null effect on these probiotics. These effects were strongly correlated with their non-bioaccessible contents: negatively for AFB1, positively for OTA, and not correlated when summing up AFB1 with OTA. As AFB1 and OTA were able to modulate the growth of two important probiotics when using isolated strains, it was necessary to confirm their effects on those probiotics as well as others using a gut microbiota approach. After 24 h colonic fermentation, although AFB1, OTA, and MIX did not impact the total bacterial content, an impact on bacterial composition was observed, confirming their impact on Lactobacillaceae and Bifidobacteriaceae families as well as on other important families belonging to Firmicutes (*Ruminococcaceae, and Lachnospiraceae*) and Bacteroidetes (*Bacteroidaceae*) phyla. These data agree with previous reports showing OTA’s ability to shift the microbiota balance, increasing Lactobacillaceae and decreasing Bacteriodaceae. At the genus level, OTA decreased the population of *Bacteroides*, *Dorea*, *Escherichia*, *Oribacterium*, *Ruminococcus*, and *Syntrophococcus*, while it increased the number of *Lactobacillus* members [34]. With regard to AFB1, although less information was found regarding its impact on the gut microbiota, this mycotoxin has been reported as being able to decrease phylogenetical diversity but increase the evenness of the community composition. Lactic acid bacteria can also be reduced in the presence of AFB1, although there are no changes at the phylum level, as reviewed by Liew and Mohd-Redzwan [34]. When using animal models, differences in gut microbiota abundances have been reported after exposure to AFB1: (i) an increase in Bacteroidetes and a decrease in Firmicutes were also verified by Gorsu [35] after 30-day exposure of piglets to an AFB1 (0.32 mg/kg)-contaminated diet, and (ii) an increase in Lachnospiraceae family abundance was also verified in the mice gut microbiota after exposure to AFB1 [36]. In addition, probiotics from both Lactobacillus and Bifidobacteria strains have been suggested as suitable binders of AFB1 and OTA, reducing their bioaccessibility [27]. Besides mycotoxin-binding capacity, the addition of *Lactobacillus casei* Shirota in feed contaminated with AFB1 had ameliorative effects on gut microbiota changes induced by AFB1 [23] as increased microbiota richness.

Regarding the impact of non-bioaccessible mycotoxins on intestinal cells’ viability and inflammation, the fecal supernatant resulting from colonic fermentation of AFB1, OTA, and MIX meals did not trigger the NF-kB activation pathway after exposure of HT29 reporter cells, suggesting that the immune system is not compromised by non-bioaccessible mycotoxins. NF-kB is a transcription factor that plays a major role in the regulation of genes responsible for inflammatory and immune responses [37]. Previous studies have shown that mycotoxins such as patulin and deoxynivalenol are able to suppress NF-kB-mediated activation via Toll receptor signaling [38,39], which could compromise the immune system, weakening the response to pathogens. These results suggest that the gut microbiota is able to reduce mycotoxins’ toxicity.

In summary, if a meal is contaminated with a single mycotoxin, in the case of AFB1, 50% bioaccessible AFB1 will have a strong impact on intestinal cells’ viability and the other 50% non-bioaccessible AFB1 on specific probiotic strain growth (*L. casei* and *B. lactis*). In the case of OTA, since a higher value will be bioaccessible (~70%), it will trigger NO production at the intestinal level, and the non-bioaccessible fraction will have a less impact on probiotic growth. In a co-exposure situation, higher contents of AFB1 will become bioaccessible, potentiating loss of intestinal viability and increased NO production at the intestinal level, but the impact on probiotic growth will be neglected. After colonic fermentation, the fecal supernatant did not trigger the NF-kB activation pathway, suggesting that the gut microbiota is able to reduce mycotoxins’ toxicity.

## 4. Materials and Methods

### 4.1. Sample Preparation and Spiking

Liquid yogurt and corn cookies were obtained from local supermarkets in Porto (Portugal). Snack meals were prepared by mixing 1 yogurt (160 g) with 2 corn cookies (16 g), representing 91 and 9% of the meal composition, respectively. Prior to meal preparation, the corn cookies were ground for 12 s at 8000 rpm (Grindomix GM 200; Retsch GmbH, Haan, Germany) and then the respective amount (16 g) mixed with the yogurt. The total amount of meal (160 g) was used as a real meal proportion to allow downscaling to an in vitro approach using 10 g of the sample.

The same meal preparation (160 g) was used for all experiments to guarantee the same meal nutritional composition, appearance, and consistency. Thus, 30 g of the meal was used for each condition and contaminated with the respective amounts of mycotoxins: (a) control (without mycotoxins), (b) contamination with 9 µg of AFB1, (c) contamination with 9 µg of OTA, and (d) simultaneous contamination with 6 µg of AFB1 and 6 µg of OTA. Duplicates of gastrointestinal digestion were assessed for each condition. After contamination, the real content of mycotoxins was quantified, as described in Section 4.3. Thus, each 10 g of the sample used for digestion had 3.0 ± 0.2 µg of AFB1 or 3.2 ± 0.2 µg of OTA for single exposure and a combination of 2.2 ± 0.3 µg of AFB1 and 2.0 ± 0.2 µg of OTA for the co-exposure situation. Although this level of mycotoxins is higher than the amount usually found in foods, it was selected to counterbalance the dilution factor of digestion experiments and allow the quantification of mycotoxins at all steps of digestion (all gastric and intestinal emptyings). The samples were stored at −20 °C in plastic bags until being used for digestion experiments. Mycotoxin standards OTA (1 mg, purity 97%) and AFB1 (1 mg, purity > 98%) were purchased from Sigma-Aldrich (St. Louis, MO, USA), and individual stock solutions of 5 µg/mL were prepared and used for artificial contamination purposes.

### 4.2. In Vitro Upper Gastrointestinal Digestion

Upper gastrointestinal digestion composed of oral, gastric, and duodenal phases was performed according to the standardized semi-dynamic protocol described by Mulet-Cabero et al. [10]. The energy value of the meals was calculated based on the macronutrient content found in the package: the yogurt and the corn snacks had 1.6% and 1.9% fat, 3.8% and 8.1% protein, and 10% and 81.6% carbohydrates, respectively. A proportion of 1 yogurt (160 g) plus 2 cookie (16 g) snacks was defined for the study. This proportion resembled a real meal of 176 g and was scaled down using the protocol’s supplementary information. The enzymes (pepsin and pancreatin extract) and the bile extract used in this study were purchased from Sigma-Aldrich. The enzymatic activities were determined prior to digestion experiments, as recommended by Mulet-Cabero, et al. [10].

Briefly, 10 g of the snack meal was mixed with simulated salivary fluid (2.00 mL simulated salivary electrolyte fluid (eSSF), 12.5 µL of 0.3 M CaCl_2_(H_2_O)_2_, and 0.363 mL of ultrapure water) using human saliva as an amylase source (150 U/mL of SSF) for 2 min at 37 °C. Then, the bolus (12.5 mL) was transferred into a 70 mL glass v-form vessel thermo-stated at 37 °C containing 10% of the simulated gastric fluid corresponding to *in vivo* fasting conditions. The remaining 90% of the simulated gastric fluid (8.75 mL of simulated gastric electrolyte fluid (eSGF), 0.925 mL of 1.5 M HCl, 6.3 µL of 0.3 M CaCl_2_(H_2_O)_2_, 1.57 mL of ultrapure water, and 1.125 mL of enzyme solution (4000 U pepsin/mL SGF)) was added at a constant ratio by separated devices. A printed paddle stirrer was used at 15 rpm for agitation. Five gastric emptyings (GEs) of 5 mL each were performed every 17 min. The gastric phase had a total time of digestion of 85 min. Before each gastric emptying, the vessel content was mixed to make the sampling more accurate. The pH was measured in each emptied aliquot, and the pH was raised to 7 with NaOH 1N (300 µL) to inactivate pepsin and prepare samples for the intestinal phase. The tubes were centrifuged at a low rotation (2000× *g*, 5 min) to obtain a 1 mL aliquot for mycotoxins analysis in the gastric bioaccessible phase. The remaining amount was kept at 4 °C for intestinal digestion the following day.

The intestinal phase was performed using a static methodology according to the INFOGEST 2.0 protocol [40]. Briefly, to 4.3 mL of neutralized chyme of each GE, 3.7 mL of simulated intestinal fluid (2.5 mL of simulated intestinal electrolyte fluid (eSIF), 8.0 µL of 0.3 M CaCl_2_(H_2_O)_2_, 0.492 mL of ultrapure water, 0.200 mL of pancreatin solution (200 U trypsin/mL SIF), and 0.500 mL of bile extract (20 mmol/L SIF)) was added and incubated at 37 °C for 2 h under agitation (15 rpm). After digestion, the samples were immediately immersed in ice for 10 min, followed by centrifugation at 10,000× *g* for 5 min at 4 °C. Both bioaccessible (BIO) and non-bioaccesible (NBIO) fractions were collected. Mycotoxin extraction from the BIO fraction was performed on the same day of digestion and the remaining digested volume stored at −20 °C until further analysis. The NBIO fraction was stored at −80 °C until used for colonic fermentation assay.

### 4.3. Mycotoxin Analysis

To 200 µL of digested samples, 4 µL of the internal standard (IS,10 µg/L of aflatoxin B2, purity 98%, Sigma-Aldrich) was added and vortexed. Then, the sample was mixed with an equal volume (200 µL) of extracting solvent (ACN, w/2% formic acid). Afterward, 50 mg of MgSO_4_ and 35 mg of NaCl were added, vortexed immediately, and centrifuged at 10,000× *g* for 5 min. The organic phase was collected in a 2 mL vial, evaporated to dryness under a N_2_ stream, and re-dissolved in 60 µL of extracting solvent. The extraction of mycotoxins in the solid samples (before digestion) was performed in the same way but with two additional steps: to 500 mg of the samples, 4 µL of IS (10 µg/L) was added and left to homogenize for 30 min in an orbital shaker. Then, 1 mL of ultrapure water was added and homogenized for another 30 min. Thereafter, the extraction followed the same procedure, as mentioned above. In the end, 20 µL of the samples were injected into an HPLC system (Jasco LC PU-1580, Tokyo, Japan) with an auto-sampler (Jasco AS-2057 Plus, Japan), a YMC-triart C18 ExRS column (150 × 3.0 mm, 3 µm, 80 Å), and a pre-column from Phenomenex (C18, 4 × 3.0 mm (i.d.), with the column kept at 40 °C. The mycotoxins were eluted using a flow rate of 0.5 mL/min of acidified water (2% formic acid, eluent A) and acidified acetonitrile (2% formic acid, eluent B) as mobile phases, with the following gradient: 30% B in the first 2 min, increasing to 95% B in 10 min, holding this condition for 5 min, and then decreasing to 30% B in 4 min, followed by 5 min of stabilization (total run time of 26 min). AFB1, AFB2, and OTA were detected by fluorescence (FLD, Jasco FP-920, Japan) using excitation/emission wavelengths of 365/455 nm and 333/465 nm for AFs and OTA, respectively. Borwin version 1.5 Data Software (JMBS, France) was used to analyze and export the data.

The method was evaluated using an in-house validation approach assessing linearity, accuracy, and precision to perform reliable quantitative results according to EU guidelines and ICH recommendations [41,42]. The matrix effect was also evaluated for both mycotoxins according to SANTE guidelines [28].

### 4.4. Impact of Bioaccessible Mycotoxins on Caco-2 Cells’ Viability and on Inflammation

Caco-2 cells were grown in complete medium (CM) composed of Dulbecco’s Modified Eagle Medium (DMEM), 10% fetal bovine serum (FBS), 1% glutaMAX, 1% non-essential amino acids (NEAAs), and 1% penicillin/streptomycin (all purchased from Gibco/Life Technology, Paisley, United Kingdom) at 37 °C with 5% CO_2_. When the cells were 80% confluent, they were trypsinized and seeded in 96-well plates (5 × 10^4^ cells/mL) and left for differentiation over 7 days with medium renewal every 2 days. For these assays, the 5 intestinal gastric emptyings were combined to obtain the total mycotoxins available after the intestinal phase. Then, Caco-2 cells were exposed to the digested sample (12× diluted in CM) for 3 h. After the exposure time, the cells were used for ROS determination, while the cells supernatant was used for NO, as described by Pinho et al. [43]. The viability of Caco-2 cells was also determined by measuring the release of lactate dehydrogenase in the cells’ supernatant according to Kaja et al. [44]. The pro-inflammatory effect of bioaccessible mycotoxins (BIO fraction) was carried out by measuring as a marker of inflammation nitric oxide (NO) and reactive oxygen species (ROS) formation on 7-day-differentiated Caco-2 cells [43,44].

### 4.5. Effect of Non-Bioaccessible Mycotoxins on Probiotics Lactobacillus casei and Bifidobacterium lactis Growth

The effect of mycotoxins that remain in the NBIO fraction after in vitro digestion was checked in two beneficial bacterial strains, *Lactobacillus casei* BL23 and *Bifidobacterium lactis* ATCC 27536, to infer the impact of mycotoxins on bacterial growth and choose the ideal proportion of the NBIO fraction for colonic fermentation, given their relevance for the human gut microbiota [45]. These were grown in MRS + 0.05% L-cysteine under anaerobic and static conditions for 24 h at 37 °C in the presence or absence of 20 µL of the sample according to Alcántara et al. [46]. Changes in optical density at 595 nm were registered in a POLARStar reader (BMG Labtech, Ortenberg, Germany), and the strains’ growth data were modeled using the Gompertz equation [47] in order to mathematically describe the microbial growth and compute the specific growth rate in the exponential phase and optical density in the stationary one.

### 4.6. In Vitro Colonic Fermentation

The NBIO fraction resulting from the duodenal phase followed in vitro colonic fermentation to evaluate the impact of non-bioaccessible mycotoxins on the microbiota and intestinal function. First, a pool of feces from healthy human volunteers (*n* = 5) was gathered and the fecal microbiota was extracted with saline solution (1:10 proportion (*w*/*v*)), homogenized in an orbital shaker over 30 min, and separated from the rest of the fecal material using a Nycodenz^®^ density gradient without affecting the original microbiota composition [48]. Afterward, the fecal microbiota was transferred to colonic fermentation tubes along with sterile basal medium (as described by [49]) and left overnight at 37 °C under anaerobic conditions for stabilization (~24 h). The following day, the NBIO fraction (2%, *w*/*v*) was added to the colonic fermentation tubes and left for 24 h under static anaerobic conditions. Duplicates of colonic fermentation were assessed for each sample. At each time point, both supernatant and pellet were collected for further analysis. The quantity and profile of bacteria before colonic fermentation were also assessed.

### 4.7. Total Bacteria Quantification by qPCR and 16S rRNA Amplicon Sequencing

The DNA was extracted according to fecal-sample-based adaptation to the Maxwell^®^ RSC PureFood GMO and Authentication Kit (Catalo No-AS1600; Promega Corporation, Madison, WI, USA). Briefly, the pellets resulting from colonic fermentation were homogenized with 850 µL of CTAB buffer and transferred to 2 mL tubes together with beads for further lysis using the FastPrep-24 5G homogenizer for 2 cycles at 6.0 m/s for 60 s, with a 30 s pause (MP Biomedicals). The samples were then heated at 95 °C for 5 min (vortex at half time), followed by the addition of Proteinase K (40 µL) and RNAase (20 µL), vortexed, and heated at 70 °C for 10 min. Then, the samples were centrifuged at 13,000 rpm for 5 min. Maxwell^®^ RSC Cartridge preparation and loading occurred, as detailed by the manufacturer. All samples were eluted in 50 μL of the provided elution buffer. The extracted DNA was quantified (ng/µL) using a Qubit 2.0 fluorometer (Life Technology) before storing at −80 °C until further use.

Quantification of total bacteria was carried out by quantitative PCR through amplification and detection of the 16S rRNA gene. In each 96-well plate (Sorenson BioScience, Inc., Murray, UT, USA), 10 µL of the reaction mixture was created by 3.5 µL of PCR water, 0.25 µL of the forward primer (10 µM, 515F (5′-GTGCCAGCMGCCGCGGTAA), 0.25 µL of the reverse primer (10 µM, 789R (5′-GCGTGGACTACCAGGGTATCT), 5 µL of the Light Cycler 480 SYBR Green I Master mix (Roche Life Science), and 1 µL of the sample, standard, or water (negative control) (all performed in duplicate) [50,51]. The Light Cycler 480 Real-Time PCR System (Roche Life Science) was used to amplify the DNA as follows: 95 °C for 10 s, 62 °C for 10 s, and 72 °C for 20 s over 40 cycles, followed by dissociation curve analysis. The standard curve used to calculate the bacterial concentration in each sample was obtained by using tenfold dilutions of specific DNA fragments.

DNA was also used for the bacterial taxonomic composition by 16S rRNA gene sequencing of the V3–V4 region (Illumina platform using a paired-end 2 × 250 bp reading system). Raw sequences were processed in-house by Novogene Bioinformatics Technology Co. Ltd. (Tianjin, China). In brief, paired-end reads were assigned to samples based on their unique barcodes and truncated by cutting off the barcodes and primer sequences. Paired-end reads were merged using FLASH (V1.2.7) [52] (see details at [53]), a fast and accurate analysis tool, which is designed to merge paired-end reads when at least some of the reads overlap the read generated from the opposite end of the same DNA fragment, and the splicing sequences were called raw tags. Quality filtering on the raw tags was performed under specific filtering conditions to obtain high-quality clean tags [54] according to the QIIME (V1.9.0) [55] quality-controlled process. The tags were compared with the reference database (SILVA database, see [56]) using the UCHIME algorithm [57] to detect chimera sequences, and then the chimera sequences were removed. Sequences with ≥97% similarity were assigned to the same operational taxonomic unit (OTU), and taxonomy was assigned with the SILVA database (see details at [56]) for microbial annotation at each taxonomic level (phylum, family, genus levels).

### 4.8. Impact of Non-Bioaccessible Mycotoxins on HT29 NF-kB Reporter Cells’ Viability and on Inflammation

The pro-inflammatory effect of in vitro colonic fermentation supernatants was carried out, as detailed by Alcántara et al. [46], with some modifications. Briefly, the pro-inflammatory effect of mycotoxins after colonic fermentation of the NBIO fraction was determined using a stably transfected HT29 reporter cell line to measure secreted embryonic alkaline phosphatase (SEAP) activity upon activation of the NF-kB pathway. HT29 reporter cells were grown in DMEM supplemented with 10% FBS, 1% (*v*/*v*) sodium pyruvate, 1% P/S, and zeocin (200 µg/mL) at 37 °C and 5% CO_2_. For this experiment, HT29 cells were seeded in 96-well plates at 6.5 × 10^4^ cells/well and incubated at 37 °C and 5% CO_2_ for 24 h. The following day, the cells were exposed to fecal supernatants in a 1:10 dilution for 24 h. After incubation, the cell supernatant was collected and SEAP activity measured using p-nitrophenyl phosphate as a phosphatase substrate according to the manufacturer’s instructions (Thermo Fisher Scientific, ref. 37620). Cell viability was measured using the resazurin reduction assay. Briefly, 150 µL of resazurin solution (10 µg/mL) was added to each well and incubated at 37 °C and 5% CO_2_ for 5 h so that viable cells could reduce resazurin to fluorescent resorufin, whose amount is directly proportional to the number of living cells, and cell viability was measured at 540–15 nm excitation and 590–20 nm emission using a CLARIOstar (BMG Labtech) plate reader.

### 4.9. Statistical Analysis

Statistical analysis was performed and graphs created using GraphPad Prism 8.1.0 for Mac. The variables’ normality was assessed by the Shapiro–Wilk test. On those variables following normal distribution, Student’s *t*-test was applied for comparison of AFB1 or OTA bioaccessibility between isolated and mixed digestion, while the Mann–Whitney U test was applied for variables not following normal distribution. For cell culture assays and total bacteria, one-way ANOVA and Dunnett’s post hoc test (parametric) or Kruskal–Wallis and Dunn’s post hoc tests (non-parametric) were applied to compare AFB1, OTA, or MIX effects in comparison with the control meal (without mycotoxins). Pearson correlations were used to test the association between the mycotoxin content in the meal, the bioaccessible fraction, and the non-bioaccessible fraction with results obtained from cell/bacterial assays (cell viability, inflammation, or bacterial growth). Pearson correlations were performed using IBM SPSS statistics software version 17.0.

## Figures and Tables

**Table 1:** Linearity range, regression equations, coefficients of determination (R^2^), limits of detection (LODs), and limits of quantification (LOQs) of AFB1 and OTA in the 3 analyzed matrices (meal, gastric digest, and intestinal digest).

		Linearity			LOD (ng)	LOQ (ng)
		Range (ng)	Regression Equation	R^2^		
AFB1	Meal	2–40	y = 0.0428x − 0.111	0.9978	2.2	7.3
	Gastric digest	1–20	y = 0.0479x + 0.0789	0.9963		
	Intestinal digest	1–20	y = 0.0697x + 0.145	0.9939		
OTA	Meal	2–40	y = 0.314x − 1.01	0.9931	0.76	2.52
	Gastric digest	1–20	y = 0.655x + 0.143	0.9947		
	Intestinal digest	1–20	y = 0.328x − 0.106	0.9993		

**Table 2:** Recoveries (%), intraday precision and interday precision (% RSD), of the analytical method applied to quantify mycotoxins in the digest fluids (gastric and duodenal) after meal digestion.

	Spiked Level (ng/mL)	Recovery (Interday, % RSD)	Intraday (% RSD)
AFB1	17	92 (9)	5
	34	105 (3)	8
	85	102 (10)	9
OTA	11	86 (25)	14
	22	80 (22)	19
	55	69 (15)	28

**Table 3:** Pearson correlations between AFB1, OTA, and AFB1 + OTA contents in nanograms in the meal before digestion or the bioaccessible fraction and impact on cells (viability and inflammation). Significant correlations are highlighted in bold.

	AFB1 Meal	OTA Meal	AFB1 BIO	OTA BIO	Sum BIO AFB1 + OTA	NO	ROS	LDH
AFB1 meal	**1**							
OTA meal	−0.317	**1**						
AFB1 BIO	**0.977**	−0.264	**1**					
OTA BIO	−0.431	**0.946**	−0.390	**1**				
Sum BIO AFB1 + OTA	0.345	0.731	0.404	0.685	**1**			
NO	0.298	**0.535**	0.240	0.338	**0.525**	**1**		
ROS	0.179	−0.045	0.040	−0.177	−0.144	0.692	**1**	
LDH	**0.640**	0.187	**0.637**	−0.059	**0.446**	0.793	0.579	**1**

**Table 4:** Effect of non-bioaccessible mycotoxins on the maximal optical density (MOD), growth rate, and lag time of *Lactobacillus casei* BL23 and *Bifidobacterium lactis* ATCC 27536 strains. Data are presented as the mean ± SD of four replicates (*n* = 4). Differences between meals with mycotoxins (AFB1, OTA, or MIX) in relation to the control meal are indicated as * *p* < 0.05, ** *p* < 0.01, *** *p* < 0.001, and **** *p* < 0.0001.

*Lactobacillus casei*					
NBIO		AFB1	OTA	MIX	Control
0.5%	MOD	3.128 ± 0.036	3.114 ± 0.034	3.176 ± 0.040	3.132 ± 0.023
	Growth rate (μmax) (h^−1^)	0.4502 ± 0.014	0.4574 ± 0.014	0.453 ± 0.015	0.4735 ± 0.010
	Lag time (h)	5.29 ± 0.137	5.228 ± 0.131	5.326 ± 0.149	5.298 ± 0.088
1%	MOD	3.23 ± 0.038	3.235 ± 0.024	3.122 ± 0.052 *	3.202 ± 0.032
	Growth rate (μmax) (h^−1^)	0.3527 ± 0.009 ****	0.5211 ± 0.011 ***	0.3659 ± 0.014 ****	0.4555 ± 0.012
	Lag time (h)	4.92 ± 0.147 **	5.44 ± 0.084	4.863 ± 0.210 **	5.39 ± 0.121
2%	MOD	2.364 ± 0.169 ***	3.104 ± 0.026 **	2.946 ± 0.061	2.924 ± 0.059
	Growth rate (μmax) (h^−1^)	0.131 ± 0.006 ****	0.3696 ± 0.007 ****	0.228 ± 0.006	0.24 ± 0.007
	Lag time (h)	1.141 ± 1.049 ***	5.383 ± 0.103 **	4.308 ± 0.258	4.398 ± 0.256
***Bifidobacterium lactis***					
**NBIO**		**AFB1**	**OTA**	**MIX**	**Control**
0.5%	MOD	7.078 ± 0.025 *	6.788 ± 0.041 ***	7.013 ± 0.030	7.014 ± 0.026
	Growth rate (μmax) (h^−1^)	0.7646 ± 0.006	0.7319 ± 0.009	0.7569 ± 0.007	0.764 ± 0.006
	Lag time (h)	5.858 ± 0.044	5.808 ± 0.074	5.844 ± 0.053	5.88 ± 0.046
1%	MOD	7.078 ± 0.025 *	6.788 ± 0.041 ****	7.013 ± 0.030	7.014 ± 0.026
	Growth rate (μmax) (h^−1^)	0.7646 ± 0.006	0.7319 ± 0.009 **	0.7569 ± 0.007	0.764 ± 0.006
	Lag time (h)	5.858 ± 0.044	5.808 ± 0.074	5.844 ± 0.053	5.88 ± 0.046
2%	MOD	5.585 ± 0.042 ****	6.224 ± 0.058 ***	6.095 ± 0.087	5.978 ± 0.050
	Growth rate (μmax) (h^−1^)	0.5585 ± 0.008 ****	0.6715 ± 0.013	0.6742 ± 0.021	0.6902 ± 0.013
	Lag time (h)	5.689 ± 0.092 ****	6.281 ± 0.115 *	6.289 ± 0.173	6.515 ± 0.101

**Table 5:** Pearson correlations between AFB1, OTA, and AFB1 + OTA contents in nanograms in the meal or the non-bioaccessible fraction and growth curve parameters of *L. casei* and *B. lactis* strains. Significant correlations are highlighted in bold.

		AFB1 Meal	OTA Meal	AFB1 NBIO	OTA NBIO	Sum NBIO AFB1 + OTA	*L. casei*			*B. lactis*		
							MOD	Growth Rate	Lag Time	MOD	Growth Rate	Lag Time
	AFB1 meal	1										
	OTA meal	−0.309	1									
	AFB1 NBIO	0.954	−0.424	1								
	OTA NBIO	0.044	0.739	−0.181	1							
	Sum NBIO AFB1 + OTA	0.772	0.256	0.629	0.65	1						
*L. casei*	MOD	**−0.765**	**0.641**	**−0.874**	**0.556**	−0.236	1					
	Growth rate	**−0.8**	**0.786**	**−0.839**	0.437	−0.304	0.871	1				
	Lag time	**−0.792**	**0.63**	**−0.889**	**0.516**	−0.279	0.998	0.878	1			
*B. lactis*	MOD	**−0.686**	**0.733**	**−0.817**	**0.67**	−0.102	0.969	0.896	0.957	1		
	Growth rate	**−0.753**	0.373	**−0.883**	0.431	−0.342	0.919	0.695	0.912	0.883	1	
	Lag time	**−0.772**	0.216	**−0.868**	0.271	−0.457	0.863	0.614	0.86	0.798	0.981	1
